# Let’s Eliminate Diseases, Not Institutes: The Case for the Fogarty International Center

**DOI:** 10.4269/ajtmh.17-0601

**Published:** 2017-09-07

**Authors:** Philip J. Rosenthal, Karen A. Goraleski, N. Regina Rabinovich, Patricia F. Walker

**Affiliations:** Authors are members of the American Society of Tropical Medicine and Hygiene's Executive Committee; 1 Editor-in-Chief;; 2 Executive Director;; 3 President-elect;; 4 President

The National Institutes of Health (NIH) is the primary funder of medical research at federal, state, and academic institutions in the United States. With an annual budget of $32 billion, it is a primary force establishing the premier position of American medical researchers in the world and has led to countless medical advances associated with the increase in global life expectancy from about 30 years in 1900 to more than 70 years today. A significant factor contributing to this improvement has been the control of many infectious diseases, from diphtheria to polio to smallpox. Despite profound advances, this job is obviously not done. Old scourges still lurk. Tuberculosis, malaria, influenza, and numerous other infections still afflict huge numbers and kill millions each year. Vaccine-preventable diseases such as measles and diphtheria have made comebacks in some areas of the world. Pathogens identified over recent decades, including the viruses that cause AIDS, Ebola, and Zika; the bacteria that cause Lyme disease, *Clostridium difficile* colitis, and *Helicobacter*-related peptic ulcer disease and cancer; and drug-resistant bacteria and protozoans, emerge frequently to cause terrible suffering. But, there is hope. This is an exciting time to work on global infectious diseases. New diagnostics, drugs, vaccines, and other measures for control and treatment of leading infections are available or on the way. We can foresee movement from effective control to eradication of a range of terrifying diseases. We have already eradicated smallpox. Polio and Guinea worm will be added to the list soon, and we can start to see the light at the end of the tunnel for several others.

However, the advances of humanity against killer infectious diseases are at risk. Sustained success requires long-term commitment. It is disappointing that the FY2018 presidential budget recommends an 18% cut in support for the NIH. Anything less than a fully resourced NIH will have devastating effects on the entire U.S. biomedical research enterprise. These effects will be felt across the United States, where research funding supports vibrant science and hundreds of thousands of jobs in government, universities, and other institutions. Even more disturbing are predicted impacts of budget cuts on the health of Americans and those around the world.

It was particularly noteworthy that the president’s budget took the unusual step of singling out one of the 27 institutes and centers of the NIH for complete elimination, the Fogarty International Center (FIC). Given today’s political climate, having “International” in the name of the Center may have been a tactical error. Without that label, perhaps the FIC would have flown under the radar. Be that as it may, it is informative to examine the work of the FIC and then consider the cost.

The FIC was established on July 1, 1968 by President Lyndon Johnson and named for Representative John E. Fogarty (D-RI), who served in Congress for 27 years, and championed international health research to reduce suffering and foster peace and prosperity throughout the world. During his tenure in the House of Representatives, including service as Chair of the Appropriations Subcommittee with responsibility for health funding, the budget for NIH grew from $37 million in 1949 to $1.24 billion in 1967. Over the course of his Congressional career, Fogarty argued unsuccessfully for the creation of an international health research institute. He died of a heart attack on January 10, 1967, and his untimely passing at age 53 stimulated action from the Johnson White House to create the FIC in the following year. The 50th anniversary of the Fogarty Center is coming up next year. How sad that it may not survive to celebrate this anniversary!

The FIC supports basic, clinical, and applied research and training for U.S. and international investigators working internationally. It funds more than 500 projects involving about 100 American universities. About 80% of FIC grants go to U.S. universities, and 100% of FIC grants involve U.S. scientists. The American scientists, in turn, collaborate with colleagues in numerous foreign countries, most of them in the developing world. A core function of the FIC is to train young medical scientists around the world in high quality research. These scientists are on the front-lines in the control of our most serious disease threats, and in the recognition and rapid management of emerging infectious diseases. When Ebola swept through West Africa in 2014, clinicians and researchers trained by the FIC worked to contain the epidemic, and although the disease spread rapidly, it was contained, except for a handful of cases, before it had the chance to spread to other regions of the world. Related stories can be told of FIC-trained investigators leading local efforts against HIV infection, tuberculosis, malaria, Zika, and a broad array of other infectious diseases. Support from the FIC has played a pivotal role in establishing a worldwide network of experts, with trained and at-the-ready workforces around the globe, to help stem the tide of devastating infectious diseases, and to respond effectively when outbreaks occur.

The annual budget of the FIC has been about $70 million in recent years. Of the total NIH budget of $32 billion, the FIC spends about 0.2%. Of the total United States federal budget of $3.8 trillion, the FIC spends about 0.002%. Consider our valuation of spending on international research programs with how Americans value coffee. Working Americans were recently estimated to spend more than $1,000 per year on coffee. The FIC budget asks for about $4.50 annually per American. Thus, maintenance of the FIC will require spending less than 1% of what we individually pay for coffee. What a deal!

Of course, there are many worthwhile programs that cost American taxpayers very little and offer huge returns, but the case for the FIC is particularly strong. In this case, a tiny percentage of the U.S. budget helps to build up defenses against disease threats in the United States while supporting improved health around the world, with the side-benefit of enormous good will for our country. Who can argue against protecting the health of all Americans, saving lives around the world, and making our world safer, by building lifelong ties between American and international scientists? All this for less than 1% of what we spend on coffee. The case is clear: to maintain American health security and the health of the world, Congress must maintain, and ideally increase funding for the FIC.

